# Complex Interplay of Empyema and Chylothorax: A Case Report With Nutritional and Clinical Implication

**DOI:** 10.7759/cureus.101403

**Published:** 2026-01-12

**Authors:** Sofia Pouriki, Dimitrios Karagiannis, Theoni Agapitou, Asimina Rautopoulou, Zafiria Mastora

**Affiliations:** 1 1st Pathology Department, General Hospital of Thoracic Diseases “Sotiria”, Athens, GRC; 2 Clinical Nutrition, Evaggelismos Hospital, Athens, GRC

**Keywords:** chylothorax, critical care, empyema, nutrition support, parenteral nutrition

## Abstract

Empyema and chylothorax are distinct but serious pleural space complications, with the simultaneous occurrence being exceedingly rare and clinically challenging. We present the case of a 78-year-old immunocompromised male with small-cell lung cancer who developed concurrent empyema and chylothorax. The patient presented with respiratory distress and signs of sepsis, and imaging revealed pleural effusion with lung collapse. Thoracostomy yielded a large volume of milky, purulent chylous fluid positive for Streptococcus sp., confirming infected chylothorax (empyema). Management included broad-spectrum tailored antibiotics, chest tube drainage, octreotide administration, and a parenteral nutrition regimen to address nutritional losses from chyle leakage. Despite initial clinical improvement, the patient succumbed to ventilator-associated pneumonia after 20 days of hospitalization. This rare case underscores the critical importance of early diagnosis and a multidisciplinary approach, including tailored nutritional therapy, for optimizing outcomes in patients with combined empyema and chylothorax.

## Introduction

Empyema represents a severe and potentially life-threatening complication arising from infections of the lungs, most commonly secondary to pneumonia, chest trauma, or thoracic surgical procedures [1]. The incidence and spectrum of causative microorganisms vary according to the source of infection (community-acquired versus hospital-acquired pneumonia), patient age, and the presence of immunosuppression. Recognized risk factors include chronic alcoholism, human immunodeficiency virus (HIV) infection, and pre-existing pulmonary disease [2]. Despite advances in antimicrobial therapy and supportive care, empyema continues to be associated with significant morbidity and mortality, with reported death rates ranging from 7% to 11%. Patients frequently experience prolonged hospitalization, and epidemiological studies have documented a rising incidence of empyema, particularly among older adults [3].

In comparison, chylothorax accounts for approximately 3% of pleural effusion cases. It exhibits no clear predilection for age or sex and is associated with an estimated mortality rate of around 10%. The condition may lead to severe complications involving the cardiorespiratory system and disturbances in fluid and electrolyte balance [4]. Management of chylothorax primarily centers on dietary modification to address substantial nutrient losses through lymphatic leakage, complemented in selected cases by pharmacological interventions such as octreotide or somatostatin [5,6].

Early recognition of both empyema and chylothorax is essential to optimize patient outcomes. Given their potential for rapid deterioration and high mortality, these conditions pose a diagnostic and therapeutic challenge, necessitating the timely identification of the underlying etiology and the prompt initiation of targeted treatment strategies to prevent serious complications.

## Case presentation

In January 2025, a 78-year-old male was brought to the emergency department by family members with complaints of progressive dyspnea, pleuritic right-sided chest pain, and oliguria (reduced urine output). His body weight was 62 kg, his body height 170 cm [body mass index (BMI) = 21.5 kg/m^2^], and his past medical history was notable for a significant smoking history of 150 pack-years, chronic obstructive pulmonary disease (COPD), arterial hypertension, and dyslipidemia. He had a known diagnosis of stage IV small-cell lung carcinoma; the primary tumor occupied the entire right upper lobe, with confirmed metastatic malignant pleural effusion, for which he was receiving chemotherapy with carboplatin and etoposide (most recent cycle administered two days before presentation). Given his malignancy and recent cytotoxic chemotherapy, the patient was considered immunocompromised.

**Figure 1:**
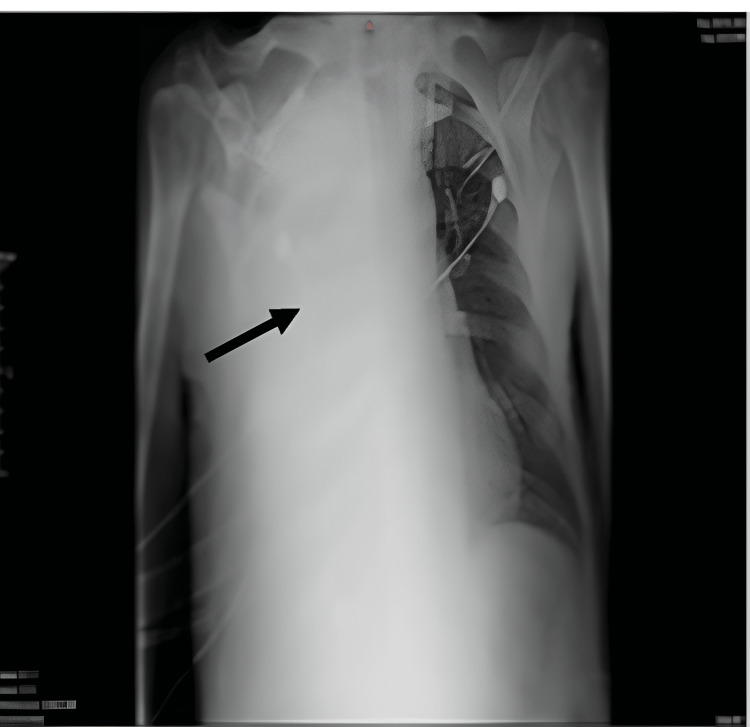
Chest x-ray indicating atelectasis of the right lung. The black arrow indicates complete opacification of the right hemothorax due to right lung atelectasis and the presence of ipsilateral pleural effusion.

**Figure 2:**
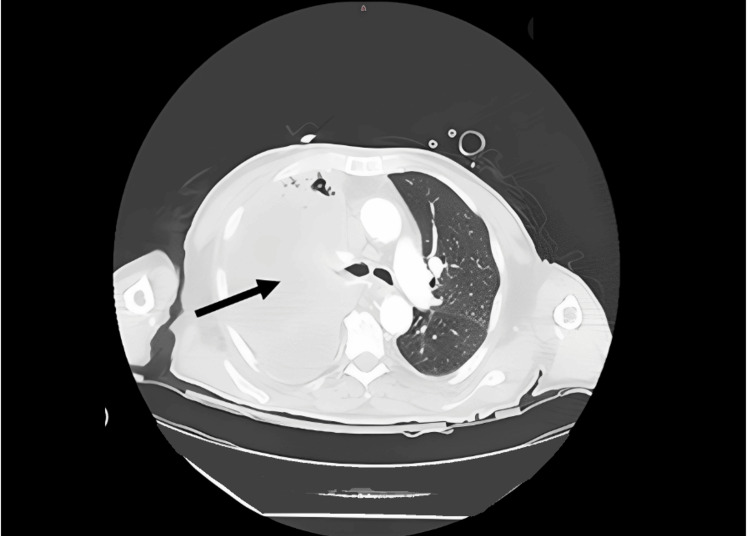
Thoracic CT. The black arrow indicates right main bronchus obstruction with secondary lobar atelectasis and pleural effusion.

**Figure 3:**
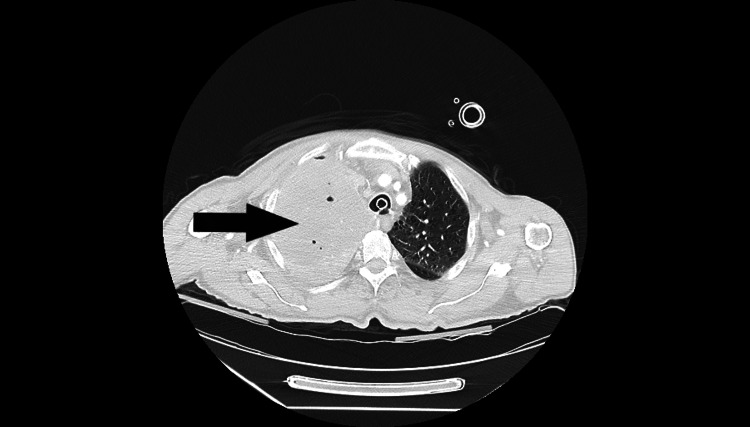
Axial CT chest section. The black arrow showing the right main bronchus obstruction.

**Figure 4:**
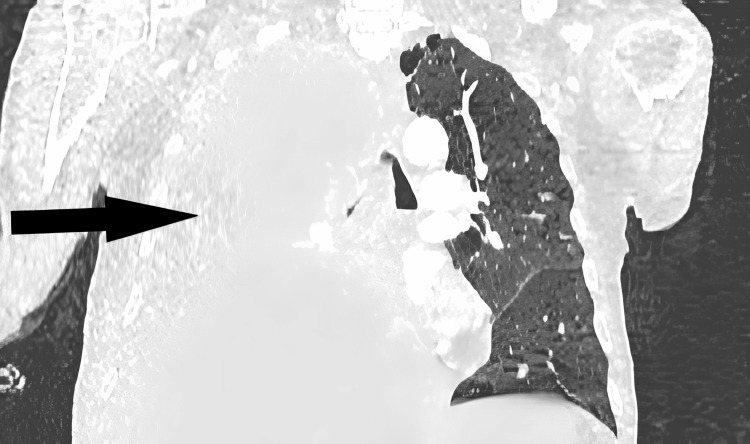
Coronal image. The black arrow shows the right main bronchus obstruction.

 Initial laboratory evaluation revealed leukocytosis [white blood cells (WBC): 23,310/mm³], thrombocytosis (platelets: 782,000/mm³), anemia (hemoglobin: 8.4 g/dL; hematocrit: 28%), and elevated inflammatory markers [D-dimers: 3.54 μg/mL; C-reactive protein (CRP): 16.57 mg/dL]. Renal function tests demonstrated acute kidney injury with creatinine of 2.3 mg/dL (baseline: 1.2 mg/dL) and urea of 263 mg/dL. Additional findings included hyperphosphatemia (8.8 mmol/L), hyponatremia (130 mmol/L), hyperkalemia (7.2 mmol/L), and mildly elevated liver enzymes [aspartate transaminase (AST): 39 IU/L; alanine aminotransferase (ALT): 55 IU/L] (Table 1). Remaining laboratory values were within normal limits. Chest radiography and thoracic computed tomography revealed complete opacification of the right hemithorax caused by a large lesion in the right lower lobe, resulting in complete obstruction of the right main bronchus and atelectasis of the right middle and lower lobes. A significant pleural effusion with cystic changes was also present, exerting mass effect on the right hemidiaphragm (Figures 1-4).

**Table 1:** Laboratory findings. WBCs: white blood cells; NEUT: neutrophils; LY: lymphocytes; HGB: hemoglobin; HCT: hematocrit; PLT: platelets; CRP: C-reactive protein; AST: aspartate transaminase; ALT: alanine aminotransferase; PCV: packed cell volume; ALP: alkaline phosphatase; Tbil: total bilirubin; γ-GT: gamma-glutamyl transferase; LDH: lactate dehydrogenase

Test	Values (Unit)	Reference Range	Test	Values (Unit)	Reference Range
WBCs	23.310/mm^3^	4.6-10.2/mm^3^	Glucose	130mg/dl	70-115mg/dl
NEUT	96%	38-68%	Urea	263mg/dl	15-45mg/dl
LY	0.9%	20-45%	Creatinine	2,3mg/dl	07-1,2 mg/dl
HGB	8.4g/dl	12.2-18.1g/dl	Sodium (Na)	130mmol/L	136-145mmol/L
HCT	28%	37.7-53.7%	Potassium (K)	7,2mmol/L	3,5-5,1mmol/L
PLT	782,000/mm^3^	140,000-450,000/mm^3^	ALP	171IU/L	28-125 IU/L
CRP	16,57mg/dL	<0,60mg/dL	Tbil	0,3g/dL	0,3-1,2g/dL
D-dimers	3,54μg/mL	0,5μg/mL	γ-GT	14IU/L	10-19IU/L
AST	39IU/L	5-40IU/L	LDH	217IU/L	135-248IU/L
ALT	55IU/L	5-35IU/L	Phosphorus	8,8mg/dl	2,6-4,5mg/dl

**Figure 5:**
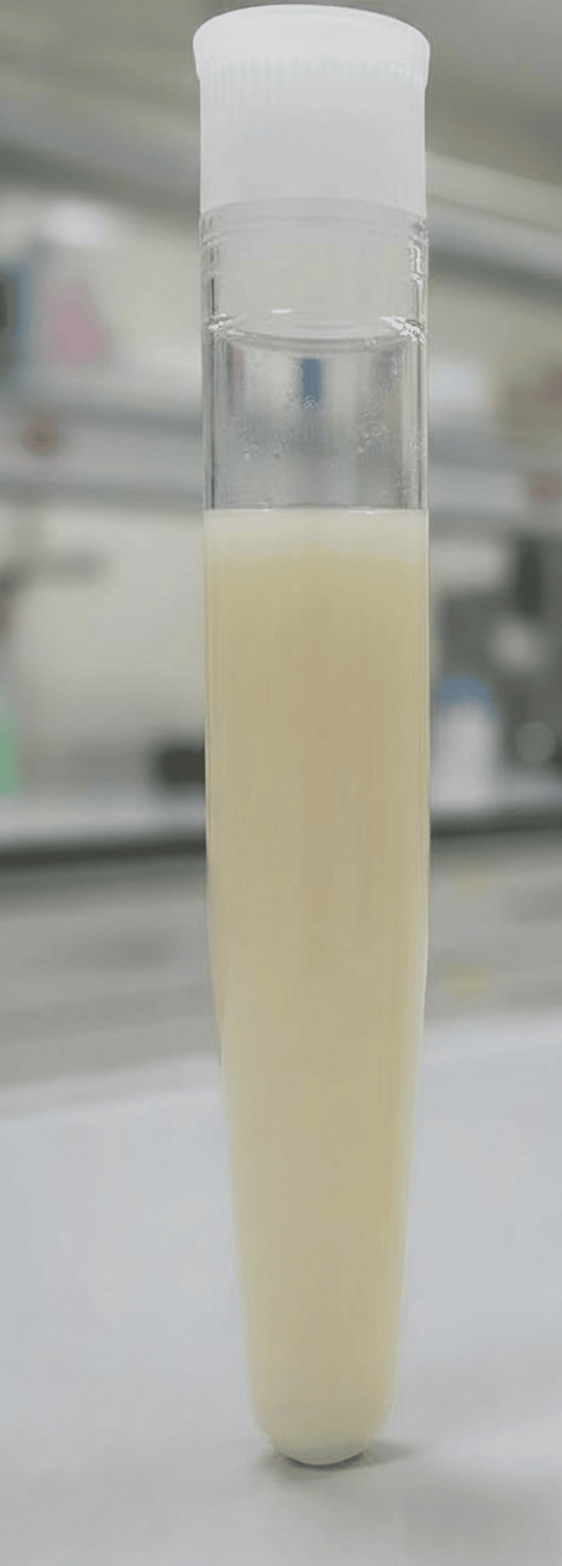
Pleural fluid gross image. Gross appearance of the aspirated pleural fluid. The specimen demonstrates a characteristic milky-white, opaque appearance, suggestive of a chylothorax.

No pulmonary embolism was detected. The patient was admitted to the internal medicine ward for severe respiratory compromise and acute kidney injury. Empirical piperacillin/tazobactam was initiated, with oxygen support via mechanical ventilation (FiO₂ 60%) and high-dose norepinephrine for septic shock. Due to deterioration in consciousness, he was intubated the same day and transferred to the intensive care unit (ICU), where sedation, vasopressor therapy (norepinephrine, vasopressin), and antibiotics were continued. Repeat chest radiography in the ICU confirmed complete fluid opacification of the right hemithorax. Thoracic surgical consultation led to chest tube placement, draining 2000ml of foul-smelling, milky fluid (Figure 5). Analysis confirmed infected chylothorax (empyema), and Streptococcus species were isolated from both pleural and blood cultures (Table 2). Comprehensive microbiological and clinical investigations were performed, including pleural fluid analysis and cultures, none of which demonstrated evidence of Mycobacterium tuberculosis. Furthermore, there were no radiological findings or clinical features suggestive of active tuberculous disease.

**Table 2:** Pleural fluid analysis.

Test	Values (Unit)
Glucose	5 mg/dl
Lactate Dehydrogenase	7318 U/L
Total Protein	1.55 g/dL
Cholesterol	75 mg/dL
Triglycerides	113 mg/dl
Albumin	0.98 U/L

**Figure 6:**
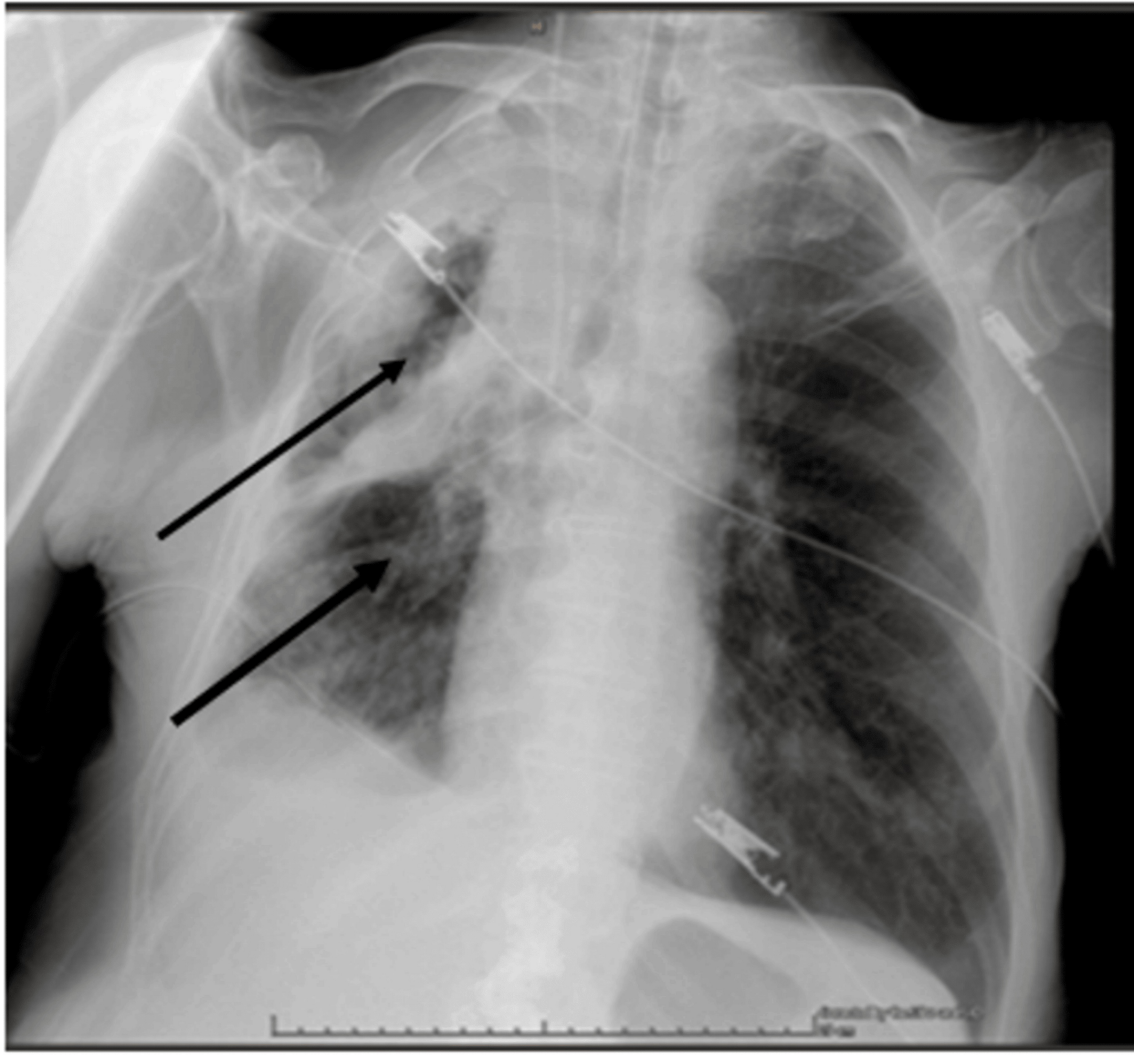
Radiological imaging of lungs. The black arrows demonstrate a significant decrease in the amount of pleural fluid, which was drained through the chest tube.

Combination therapy was empirically initiated for septic shock, comprising meropenem, vancomycin, and metronidazole, augmented by amikacin as a bactericidal agent targeting suspected bacteremia. This approach aligns with Surviving Sepsis Campaign guidelines for broad-spectrum coverage in critical illness pending culture results [7]. This clinical suspicion was subsequently confirmed by blood cultures, which isolated the identical pathogen (Streptococcus spp.) as that identified in the empyema. Concurrently, chylothorax management included octreotide use, no nutrition by mouth, initiated on total parenteral nutrition (TPN) (all in one multi-chambered bag containing intravenous lipid emulsion, in order to cover essential fatty acids and caloric requirements) alongside surgical drainage of the empyema. Nutritional targets were carefully calculated at 1800 kcal/day and 90 g protein/day (30 kcal/kg BW and 1.5 g/kg BW, respectively), based on European Society for Clinical Nutrition guidelines for critically ill patients (25-35 kcal/kg/day energy; 1.2-1.5 g/kg/day protein, using ideal BW) [8]. Close monitoring of metabolic tolerance and triglyceride levels ensured adequate energy provision while minimizing enteral lymphatic flow, supporting chylothorax resolution. Over the next 7 days, pleural drainage volumes declined significantly (<500ml/day, low output), confirmed by serial imaging (Figure 6). Surgical reevaluation advised continuation of the current regimen with close monitoring. 

The patient showed initial clinical improvement, and weaning from mechanical ventilation began. However, he developed a second episode of septic shock due to ventilator-associated pneumonia. Despite aggressive treatment, his condition deteriorated, and he died from multi-organ failure after 20 days of hospitalization.

## Discussion

Although chylothorax is an uncommon cause of pleural effusion, its impact can be profound, especially in patients who are critically ill or immunocompromised. Despite its rarity, the condition carries significant clinical and metabolic risks, and in some reports, mortality has reached as high as 80% [5,9,10]. What makes chylothorax particularly concerning is the ongoing loss of vital components such as nutrients, immunoglobulins, lymphocytes, and electrolytes. Over time, this leads to malnutrition, immune suppression, and metabolic instability [11,12]. These effects are often worse in patients with cancer, infection, or systemic inflammation, where the body’s reserves are already diminished, and catabolic processes are heightened.

Managing chylothorax is rarely straightforward. It usually requires a multidisciplinary approach, combining treatment of the underlying cause with nutritional and supportive care. The immediate goal is to reduce lymphatic leakage without allowing nutritional decline. Most clinicians begin with a very low-fat, high-protein diet, supplemented with medium-chain triglycerides (MCTs) to limit chyle flow [6]. When fat restriction must continue for a prolonged period, supplementation with fat-soluble vitamins and essential fatty acids becomes necessary, along with ensuring enough protein to prevent muscle loss. If conservative management fails, parenteral nutrition may be required, although MCT-based enteral feeding remains the preferred route whenever possible. In our case, due to a high-output chyle leak, oral intake was stopped, and total parenteral nutrition (TPN) was initiated. Interestingly, the effusion resolved completely within a week. Pharmacologic options can also help; somatostatin and its synthetic analogue octreotide have been shown to reduce gastrointestinal and pancreatic secretions, thereby lowering lymphatic flow and promoting pleural healing [9,12]. These agents are especially helpful when surgery is not an option or when standard therapy has failed. Pleural drainage remains a key supportive measure, relieving breathlessness and allowing lung re-expansion. Persistent cases may eventually require interventional radiology or surgical procedures such as thoracic duct embolization or ligation, though these are typically last-resort options [11,12].

By contrast, empyema represents a different but equally serious challenge. It usually develops after pneumonia, thoracic surgery, or trauma, and tends to affect the elderly and immunosuppressed [1,3]. Despite modern antibiotics and drainage techniques, empyema still carries high morbidity and mortality. Early recognition is essential and relies on imaging combined with pleural fluid analysis to confirm biochemical and microbiological features [13]. Once identified, treatment involves broad-spectrum antibiotics, tailored to culture results, and effective drainage through tube thoracostomy to remove pus and re-expand the lung.

The coexistence of empyema and chylothorax, as seen in this case, is exceptionally rare and can complicate both diagnosis and management. The presence of chyle can obscure purulent characteristics, making the effusion appear less obviously infectious. This can delay diagnosis and appropriate treatment. Moreover, the ongoing loss of chyle worsens the immune suppression already caused by infection, amplifying clinical deterioration. Such complex presentations highlight the importance of early, coordinated input from thoracic surgeons, infectious disease experts, intensivists, and clinical nutritionists.

## Conclusions

In summary, this case highlights how infection, malignancy, and nutritional depletion can intersect in complex pleural diseases. Early diagnosis, appropriate antibiotic therapy, timely drainage, and close nutritional management are key to success. The combination of TPN and octreotide proved particularly effective in this patient, reducing lymphatic losses and stabilizing the metabolic state. Ultimately, beyond treating the infection, attention to nutritional and metabolic balance remains essential for recovery and improved outcomes.
